# Invasive Disease Caused by Nontypeable *Haemophilus influenzae*

**DOI:** 10.3201/eid2110.150004

**Published:** 2015-10

**Authors:** Jeroen D. Langereis, Marien I. de Jonge

**Affiliations:** Radboud University Medical Center, Nijmegen, the Netherlands

**Keywords:** *Haemophilus influenzae*, sepsis, meningitis, humoral immunity, cellular immunity, virulence, pathogenicity, vaccines, bacteria, invasive disease, pneumonia, nontypeable

## Abstract

The incidence of severe *Haemophilus influenza* infections, such as sepsis and meningitis, has declined substantially since the introduction of the *H. influenzae* serotype b vaccine. However, the *H. influenzae* type b vaccine fails to protect against nontypeable *H. influenzae* strains, which have become increasingly frequent causes of invasive disease, especially among children and the elderly. We summarize recent literature supporting the emergence of invasive nontypeable *H. influenzae* and describe mechanisms that may explain its increasing prevalence over the past 2 decades.

## Abstract 2

These infections are emerging worldwide, especially in young children and the elderly.

*Haemophilus influenzae* is an extracellular bacterium that commonly colonizes the upper respiratory tract of healthy humans, who are the bacterium’s only known natural reservoir. The *H. influenzae* species is subdivided into 7 groups, including 6 that express distinct serotypes of polysaccharide capsule (a–f) and 1 unencapsulated group termed nontypeable *H. influenzae* (NTHi). NTHi is most frequently associated with mild inflammatory diseases of the human mucosa, including otitis media (OM), sinusitis, and exacerbations of chronic obstructive pulmonary disease (COPD), but it can also cause invasive disease (*1*). The incidence of invasive NTHi (usually defined as isolation of NTHi from a normally sterile site) has increased substantially since the introduction of the *H. influenzae* serotype b (Hib) vaccination in the early 1990s and of the *Streptococcus pneumoniae* polysaccharide conjugate vaccine (PCV) in the early 2000s (*2*–*5*), but factors contributing to NTHi are poorly understood. We summarize data supporting the emergence of NTHi as an increasingly prominent cause of invasive bacterial disease and propose 4 factors that may be driving its rising prevalence worldwide.

## Methods

We first summarized nationwide surveillance of invasive NTHi disease recorded by the Netherlands Reference Laboratory for Bacterial Meningitis (*6*). Next, on November 12, 2014, we systematically searched the US National Library of Medicine’s PubMed database (http://www.ncbi.nlm.nih.gov/pubmed/) by using the search terms “invasive nontypeable *Haemophilus influenzae*” and “invasive non-typeable *Haemophilus influenzae*.” We reviewed all papers published during 2000–2014 and summarized all surveillance studies meeting the following criteria: 1) written in English; 2) recording invasive *H. influenzae* cases occurring during the post-Hib vaccine era; 3) spanning >4 years; and 4) discriminating among serotype b, non–serotype b, and NTHi strains (Table; Technical Appendix). Finally, we described mechanisms that may explain increased prevalence of invasive NTHi infections over the past 2 decades.

**Table:** Invasive *Haemophilus influenzae* cases worldwide since introduction of serotype b vaccine*

Location	Period of strain collection	Surveillance method	Typing method	Changes in NTHi cases or incidence†	Serotyped Hi isolates, no.	Serotype b isolates, %	Non–serotype b isolates, %	NTHi isolates, %	Ref
Canada	1989–2007	Active, prospective surveillance	SA	Increased incidence	1,455	20	17	62	(*1*)
Canada	2000–2006	Nationwide surveillance	SA+PCR	No change	122	4	39	57	(*2*)
Europe	1996–2006	European Union Invasive Bacterial Infection Surveillance	SA or PCR	No change	7,992	35	9	56	(*3*)
Germany	2001–2004	Nationwide surveillance	Not reported	NA	147	40	14	46	(*4*)
Israel‡	2003–2012	Nationwide prospective surveillance	SA	No change	389	26	11	62	(*5*)
Multiple§	2000–2008	Active population-based surveillance	Not reported	No change	398	6	17	77	(*6*)
Portugal	2002–2010	Laboratory-based passive surveillance	PCR	Increased cases	144	13	10	77	(*7*)
Slovenia	2000–2008	National surveillance	PCR	Increased incidence	108¶	13	2	85	(*8*)
Spain	2004–2009	Nationwide surveillance	PCR	NA	307	5	8	87	(*9*)
Spain	2008–2013	Laboratory-based study	SA	NA	70	1	14	85	(*10*)
Sweden	1997–2009	Retrospective laboratory-based study	PCR	Increased cases or incidence	268#	11	18	71	(*11*)
Taiwan	1999–2002	National surveillance	SA	NA	10	20	0	80	(*12*)
USA, Alaska	1991–1996	Active surveillance	SA	NA	40	14	31	54	(*13*)
USA, Arkansas	1993–2001	Retrospective laboratory-based study	SA	NA	33	3	6	91	(*14*)
USA, Utah	1998–2008	Passive surveillance	SA	Increased cases or incidence	101	9	49	43	(*15*)
USA, Illinois	1996–2004	Passive surveillance	SA	Increased incidence	522	15	31	54	(*16*)
USA	1999–2008	Active surveillance	SA	Increased incidence	4190	4	26	70	(*17**,**18*)

*Hi, *Haemophilus influenzae*; NA, not applicable due to limited sample size (<100 isolates) or lack of year-to-year data; NTHi, nontypeable *Haemophilus influenzae*; Ref, reference (see online Technical Appendix, http://wwwnc.cdc.gov/EID/article/21/10/15-0004-Techapp.pdf); SA, slide agglutination; SA+PCR, slide agglutination positive isolates confirmed by PCR. †Increased cases = increase in number of NTHi cases in patients >1 years of age; Increased incidence = increase in NTHi incidence rate in patients >1 year of age; No change = no difference in number or incidence rate of NTHi cases. ‡Pediatric cases (<15 years of age) only. §Australia, Canada, and Denmark. ¶PCR-typed isolates from post-Hib vaccination era only. #PCR-typed isolates only.

*

## NTHi as Emerging Pathogen Causing Invasive Disease in the Netherlands

**Figure 1:**
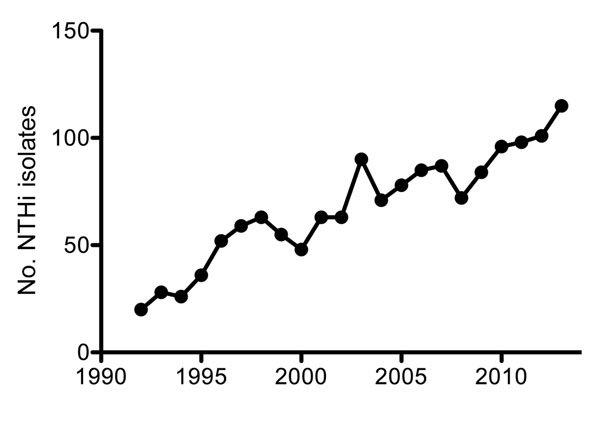
Number of recorded nontypeable *Haemophilus influenzae* (NTHi) isolates from blood or cerebrospinal fluid in the Netherlands, by year, 1992–2013. Adapted from (*6*).

**Figure 2:**
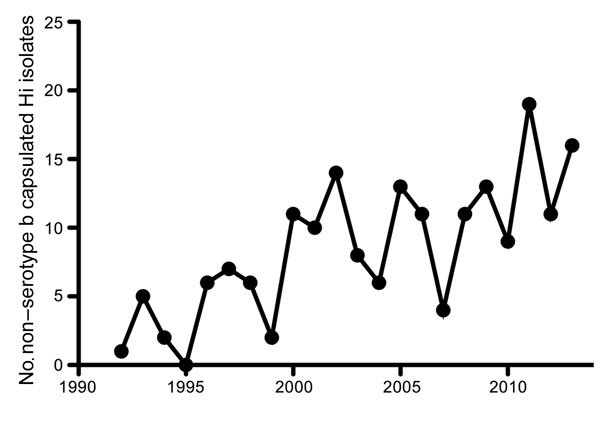
Number of recorded non–serotype b capsulated *Haemophilus influenzae* (Hi) isolates from blood or cerebrospinal fluid in the Netherlands, by year, 1992–2013. Adapted from (*6*).

Until the mid-1990s, *H. influenzae* serotype b (Hib) was a predominant cause of invasive disease (e.g., pneumonia, sepsis, and meningitis), especially in children. In 1992, a total of 294 (93%) cases with *H. influenzae* isolates that caused sepsis or meningitis in the Netherlands were attributed to Hib alone. The introduction of an effective vaccine in 1993 drastically decreased the incidence of serotype b infections, which, by 1997, represented only 19 (22%) cases with invasive *Haemophilus* isolates (*6*). However, with the near-elimination of invasive disease caused by Hib, the number of recorded invasive NTHi cases increased almost 6-fold during the past 2 decades, from 20 in 1992 to 115 in 2013 (Figure 1). Most (95%) of these NTHi isolates were collected from blood (*6*). NTHi invasion was detected mainly among persons >50 years of age (80%); Hib was found more often in children <5 years of age, who represented 45% of cases, compared with 38% for those >50 years of age (*6*). As with the increased number of invasive NTHi cases, a notable increase in the prevalence of non–serotype b encapsulated *H. influenzae* strains was also observed in the Netherlands (Figure 2), although the number of such cases remains small (*6*).

## NTHi as Emerging Pathogen Causing Invasive Disease Worldwide

Emergence of NTHi as a cause of invasive disease was reported in studies worldwide and was consistently the most prevalent *H. influenzae* that caused invasive disease (Table). In contrast, Hib cases represented <20% of invasive disease. Seven studies showed a clear increase in absolute numbers of invasive NTHi cases or increased incidence rates of invasive NTHi during the study period (Technical Appendix references *1**,**7**,**8**,**11**,**15**–**17*); 4 studies showed no increase (Technical Appendix references *2**,**3**,**5**,**6*). Large year-to-year differences in overall *H. influenzae* incidence reported by Laupland et al. make it difficult to interpret whether an increase in invasive NTHi incidence occurred (online Technical Appendix reference *6*). Furthermore, a relatively low number of patients (n = 122) over an extended collection period of 7 years complicates the year-to-year analysis in the study by Tsang et al. (Technical Appendix reference *2*). However, that study showed a significantly higher mean number of NTHi infections during 2004–2006 (12.7 ± 2.5), compared with the mean number during 2000–2003 (5.0 ± 2.6). Bamberger et al. observed no difference in the incidence of invasive NTHi, perhaps explained by the study population, which consisted of children <15 years of age (online Technical Appendix reference *5*). Globally, the average incidence of invasive NTHi is ≈1/100,000 population (*4*,*7*,*8*), a rate similar to that of the Netherlands (*9*).

Whereas Hib predominantly causes bacterial meningitis in healthy children <5 years of age, most invasive NTHi disease is found in very young children (<20 weeks of age) and the elderly (>65 years). In these populations, NTHi develops as pneumonia or bacteremia without apparent focus of infection (online Technical Appendix references *3**,**6**,**16**,**17*). These findings contrast with the widely held view that NTHi infections are mild or asymptomatic. The potential severity of invasive NTHi is illustrated by case fatality rates of 10%–20% (Technical Appendix references *3**,**6**,**16*), similar to case fatality rates for *S. pneumoniae* (*10*).

## Emergence of Capsulated, Non–Serotype b *Haemophilus influenzae* Strains

In addition to the increased incidence of NTHi infections, an increased number of invasive infections caused by encapsulated non–serotype b *H. influenzae* strains, especially Hie and Hif, has been observed in the Netherlands during the past 2 decades (Figure 2). This trend has been confirmed in multiple independent studies (Technical Appendix references *3**,**11**,**17*), although the increases are not as large as those observed for NTHi. In Europe, the incidence of non–serotype b *H. influenzae* capsulated strains was 690 (9%) of 7,992 isolates; 500 (72.5%) of the 690 isolates were Hif, and 143 (20.7%) were Hie (Technical Appendix reference *3*). This distribution of non–serotype b *H. influenzae* encapsulated serotypes causing invasive disease was similar in other parts of the world, except for specific ethnic groups where Hia is most prevalent (*11*–*13*). The epidemiology and clinical manifestations of invasive Hie and Hif strains are similar to that of NTHi and mostly occur as pneumonia or bacteremia in the elderly (*14*). In contrast, invasive Hia infections are more similar to Hib infections than to NTHi infections. Hia infections occurred mainly in young children and frequently as meningitis (*12*,*13*). The apparent similarity between Hia and Hib might be attributed to similarities in capsule structure; both contain a neutral sugar, an alcohol (ribitol), and a phosphodiester (*15*).

## Possible Explanations for Emergence of Nontypeable *Haemophilus influenzae*

Although numbers of NTHi cases are increasing, underlying mechanisms for the increase are yet to be determined. We offer 4 possible explanations for the emergence of NTHi as a pathogen causing invasive disease.

### 1) Vaccine-Mediated Strain Replacement

The success of the Hib vaccine and PCV is attributed to the strong immunogenic properties of polysaccharide conjugate formulations. However, protection against *H. influenzae* is limited to serotype b, and the possibility exists that another disease, caused by other *H. influenzae* strains against which vaccines offer no protection, may replace Hib. In fact, already in 1997, Marc Lipsitch anticipated the possibility of strain replacement with the introduction of the Hib vaccine (*16*). The significance of strain replacement remains controversial: some clinical studies highlight its potential danger (*2*,*4*); others fail to observe it altogether (*8*,*17*). Besides the introduction of the Hib vaccination, introduction of the PCV has also been proposed as contributing to *H. influenzae* strain replacement. Multiple studies show substantial increases in NTHi nasopharyngeal colonization (*3*,*5*) and in percentage of OM cases caused by NTHi in PCV-vaccinated persons (*18*–*20*). This increased nasopharyngeal carriage of NTHi in PCV-vaccinated children might increase transmission to groups susceptible to invasive NTHi disease, such as the elderly, and might thereby contribute to the emergence of invasive NTHi disease. Strain replacement during colonization of persons >65 years of age appears to lack investigation, possibly because of the relatively low percentage of nasopharyngeal carriage in this age group. However, NTHi carriage rates in parents of PCV-vaccinated children increased from 23% prevaccination to 40% postvaccination (*5*), indicating that carriage of NTHi has increased in healthy adults, possibly because of increased transmission from PCV-vaccinated children. Increased NTHi carriage might contribute to the increased number of invasive NTHi disease cases recorded during the past 20 years.

### 2) Improved Bacterial Detection and Serotyping

Whether the emergence of NTHi as a cause of invasive disease indicates an actual increase in the number of cases or results from improved detection and serotyping is difficult to assess. Bacterial culture is the gold standard for *H. influenzae* detection. However, a major disadvantage of culture is that multiple days are needed to isolate bacteria and confirm culture identity. Therefore, rapid and more sensitive real-time PCR (rtPCR) assays have been developed to shorten the time needed for identification. Several rtPCR assays that target different *H. influenzae* genes have been developed and are more sensitive for detecting the *hpd* gene than for detecting genes *ompP2* or *bexA* (detection of capsulated *H. influenzae* strains only) (*21*). Despite evidence that rtPCR-based assays provide improved detection of *H. influenzae*, all studies we summarize use bacterial culture as the detection method (Table).

Slide agglutination is the gold standard for serotyping *H. influenzae* in most laboratories, although this technique is prone to misinterpretation because of nonspecific agglutination, cross-reactions, or autoagglutination. The transition from slide-agglutination to PCR-based methods that detect capsule locus genes, such as *bexA* or *bexB*, has substantially improved the accuracy of serotyping results. For instance, Kastrin et al. recently showed that 80 isolates originally serotyped as NTHi were detected as unencapsulated by PCR, but 12 (11% of total isolates) of 28 isolates reported as capsulated by slide agglutination were shown by PCR to harbor no functional capsule genes (*7*). On the basis of PCR results, 5%–20% of strains typed by slide agglutination were mistyped as encapsulated (*7*,*22*,*23*). Although PCR detection methods apparently detect more invasive NTHi isolates than does slide agglutination, the increased detection by PCR does not explain the year-to-year increase in number of invasive NTHi cases (Table) because, within each study, similar typing techniques were used throughout the study period. However, the number of invasive NTHi cases likely is underrepresented in studies that use slide agglutination for detection.

### 3) Increased Virulence of NTHi Strains

Increased bacterial virulence as a consequence of the acquisition of novel virulence factors might also contribute to invasive NTHi disease. The natural genetic competence of NTHi enables the exchange of large pieces of DNA between strains at a high frequency (*24*), a process that supports acquisition of novel virulence factors.

Because invasive infection and death of host do not enhance transmissibility of virulent NTHi, the evolutionary basis for these genetic changes may lie in fitness advantages during nasopharyngeal colonization, a theory supported by recent studies. For instance, we have shown that NTHi isolates collected from middle ear fluid of children with OM exhibited increased resistance to complement-mediated killing compared with colonizing NTHi isolates from the nasopharynx (*25*), but we found that colonizing and OM-causing NTHi strains with a similar multilocus sequence type collected from the same patient showed no difference in complement resistance (*26*). This similarity in complement resistance for NTHi strains with a similar multilocus sequence type indicates that NTHi strains had already acquired mechanisms that increased resistance to complement-mediated killing during colonization and retained them during translocation to the middle ear cavity. These observations were corroborated by a later study that showed that most of the phase-variable genes known to modulate resistance to complement-mediated killing were regulated similarly for colonizing and OM-causing NTHi strains within the same patient (*27*).

Limited data are available on the mechanisms that underlie increased NTHi virulence in patients with invasive disease. Recently, Bajanca-Lavado et al. showed that an NTHi strain that caused endocarditis appeared highly virulent because of the expression of a second copy of the IgA protease gene (*iga*B) combined with a strong resistance to complement-mediated killing (*28*). We have shown that NTHi strains collected from patients with invasive disease more frequently incorporate galactose to heptose III in the lipooligosaccharide; this modification decreases binding of IgM and thereby increases resistance to complement-mediated killing (*29*). A study by Hällstrom et al. showed a correlation between complement resistance and disease severity but no difference in complement resistance between invasive and colonizing NTHi strains (*30*). This lack of difference in complement resistance between invasive and colonizing NTHi strains might be explained by the large proportion (41%) of patients with immune deficiencies in the invasive group, therefore potentially reducing the immunologic pressure for NTHi strains to maintain complement resistance in the bloodstream.

That the type b capsule protects *H. influenzae* from the bactericidal activity of the complement system and that this protection contributes to its invasive character are generally accepted ideas. Zwahlen et al. reported that capsule transformants showed dramatic differences in virulence (*31*). Although all capsule types were able to colonize the nasopharynx of rats, bacteremia was detected only in animals challenged with serotypes a and b and with a single animal serotype, f. The highest bacterial load was found among animals infected with serotype b. Therefore, losing the protective capsule would be expected to render *H. influenzae* unable to cause invasive disease. However, recent whole genome sequencing results showed that a few invasive NTHi isolates had a multilocus sequence type usually associated with serotype b strains (*32*). In these NTHi isolates, lack of capsule expression was related to the deletion of the *bexA* gene, whereas the remaining capsule locus was similar to that of the corresponding capsulated isolates. However, the lack of a capsule did not abrogate the ability of these particular NTHi strains to cause invasive disease, indicating that other factors besides the capsule of Hib strains contribute to invasiveness. For example, Fleury et al. found that a Hib- and Hif-specific lipoprotein PH was able to bind human factor H, resulting in increased resistance to complement-mediated killing (*33*). Identification of other genetic factors might partly explain why NTHi is found to cause invasive disease.

### 4) Demographic Changes

The epidemiology of invasive *H. influenzae* disease has changed dramatically over the past 20 years (*2*,*7*). Instead of being mainly a pediatric disease caused by Hib, formerly rare capsular serotypes (mostly Hia and Hif) and NTHi cause most of invasive *H. influenzae* cases, especially among the elderly (Table). For instance, in the United States, 78% of invasive *H. influenzae* cases among adults >65 years of age were attributed to NTHi, with an even higher frequency (89%) among those >85 years of age (*34*).

Reasons for this apparent increase in susceptibility to invasive NTHi infections in the elderly are unknown, but the immunologic status of the host is believed to play a role. Coexisting conditions or risk factors such as coronary artery disease, congestive heart failure, and smoking were more common in patients with invasive disease compared with the general population (*34*). The number of patients with COPD, the third leading cause of death worldwide (*35*), is increasing. NTHi is often found colonizing the lungs of patients with COPD, and the increased number of patients with COPD might contribute to the increased incidence of invasive NTHi cases. Serum IgG levels to *H. influenzae* protein D showed a tendency to decline with age but were even lower in adults with coexisting conditions such as COPD, cancer, chronic renal failure, or diabetes, compared with age-matched healthy persons (*36*). The absence of naturally acquired antibodies against protein D, a highly conserved antigen, may contribute to increased susceptibility to invasive NTHi disease. However, invasive NTHi infections are found not only in persons with immunocompromising conditions (e.g., chronic lymphatic leukemia or multiple myeloma) or coexisting conditions (e.g., COPD, diabetes, or cardiovascular diseases) but in almost half of cases in persons who were otherwise in good health (Technical Appendix references *6**,**11*).

Recently, several groups have found that binding of IgM to the bacterial surface might play a role in the innate defense against NTHi infections (*25*,*29*,*37*). This finding is corroborated by a clinical study in which Micol et al. showed that patients with hyper-IgM syndrome were less susceptible to NTHi colonization, a finding that emphasizes the role of IgM in the immune defense against this pathogen (*38*). The percentages of IgM-producing CD27+ memory B cells in the peripheral blood of children are low but increases to almost 20% in adults and declines again in the elderly (*39*). These findings correspond with levels of susceptibility to bacterial infections such as NTHi in young children and the elderly. Studies examining serum immunoglobulin levels in patients with invasive NTHi disease compared with those of healthy age-matched patients could help address the question of whether a diminished protective immunoglobulin level in the elderly contributes to susceptibility to invasive NTHi disease.

Besides impaired humoral immunity, diminished cellular immunity has been described in the elderly. Evidence exists for a broad, age-related alteration in the development and function of lymphocytes, monocytes, macrophages, and neutrophils (*40*), although specific effects of these changes on susceptibility to invasive NTHi infections have not been investigated in detail. Recently, we showed that neutrophils efficiently phagocytose and kill opsonized NTHi bacteria (*29*), but decreased neutrophil phagocytic capacity among the elderly may impair this host defense and contribute to poorer clinical outcomes during NTHi infection.

## Conclusions

From examination of the available literature, we conclude that invasive NTHi disease is emerging worldwide and demands implementation of effective prevention. Development of vaccines against NTHi is considered paramount because this pathogen is also often found to cause pneumonia in patients with COPD and OM in children. However, development of an effective vaccine for risk groups demands knowledge about factors that contribute to the emergence of invasive NTHi disease. Age and coexisting conditions are likely predisposing factors for invasive NTHi infections. Also, increased NTHi colonization in children might contribute to increased transmission to persons susceptible to developing invasive NTHi disease. In view of these factors, broad vaccination strategies for the general public could be effective by decreasing transmission, bolstering herd immunity, and protecting potentially susceptible persons.

**Technical Appendix.** Results of systematic search of US National Library of Medicine’s PubMed database for articles on invasive nontypeable *Haemophilus influenzae* published during 2000-2014.
