# Supplementary material for: Invasive Disease Caused by Nontypeable Haemophilus influenzae
Source: Emerg Infect Dis. 2015 Oct;21(10):1711–8. doi: 10.3201/eid2110.150004 (PMC4593434; doi:10.3201/eid2110.150004)
Supplement: Supplementary file 1 — Technical Appendix. Results of systematic search of US National Library of Medicine’s PubMed database for articles on invasive nontypeable Haemophilus influenzae published during 2000-2014. [file 15-0004-Techapp-s1.pdf]

Article DOI: http://dx.doi.org/10.3201/eid2110.150004

Invasive Disease Caused by Nontypeable *Haemophilus influenzae*

Technical Appendix

References

1. Adam HJ, Richardson SE, Jamieson FB, Rawte P, Low DE, Fisman DN. Changing epidemiology of invasive Haemophilus influenzae in Ontario, Canada: evidence for herd effects and strain replacement due to Hib vaccination. Vaccine. 2010;28:4073–8. PubMed http://dx.doi.org/10.1016/j.vaccine.2010.03.075

2. Tsang RS, Sill ML, Skinner SJ, Law DK, Zhou J, Wylie J. Characterization of invasive Haemophilus influenzae disease in Manitoba, Canada, 2000–2006: invasive disease due to non-type b strains. Clin Infect Dis. 2007;44:1611–4. PubMed http://dx.doi.org/10.1086/518283

3. Ladhani S, Slack MP, Heath PT, von Gottberg A, Chandra M, Ramsay ME, et al. Invasive Haemophilus influenzae disease, Europe, 1996–2006. Emerg Infect Dis. 2010;16:455–63. PubMed http://dx.doi.org/10.3201/eid1603.090290

4. Sandqvist A, Kalies H, Siedler A, Grondahl B, Schmitt HJ, Schweitzer-Krantz S, et al. Invasive nontypeable Haemophilus influenzae infections in Germany: a case report of a previously healthy 7-year-old boy with an intracranial abscess, and epidemiological data from 2001 to 2004. Eur J Pediatr. 2006;165:658–9. PubMed http://dx.doi.org/10.1007/s00431-006-0131-2

5. Bamberger EE, Ben-Shimol S, Abu Raya B, Katz A, Givon-Lavi N, Dagan R, et al. Pediatric invasive Haemophilus influenzae infections in Israel in the era of Haemophilus influenzae type b vaccine: a nationwide prospective study. Pediatr Infect Dis J. 2014;33:477–81. PubMed http://dx.doi.org/10.1097/INF.0000000000000193

6. Laupland KB, Schonheyder HC, Ostergaard C, Knudsen JD, Valiquette L, Galbraith J, et al. Epidemiology of Haemophilus influenzae bacteremia: a multi-national population-based assessment. J Infect. 2011;62:142–8. PubMed http://dx.doi.org/10.1016/j.jinf.2010.11.009

7. Bajanca-Lavado MP, Simoes AS, Betencourt CR, Sa-Leao R.; The Portuguese Group for Study of Haemophilus influenzae invasive infection. Characteristics of Haemophilus influenzae invasive isolates from Portugal following routine childhood vaccination against H. influenzae

serotype b (2002–2010). Eur J Clin Microbiol Infect Dis. 2014;33:603–10. PubMed http://dx.doi.org/10.1007/s10096-013-1994-6

8. Kastrin T, Paragi M, Kolman J, Cizman M, Kraigher A, Gubina M, et al. Characterisation of invasive Haemophilus influenzae isolates in Slovenia, 1993–2008. Eur J Clin Microbiol Infect Dis. 2010;29:661–8. PubMed http://dx.doi.org/10.1007/s10096-010-0910-6

9. García-Cobos S, Arroyo M, Perez-Vazquez M, Aracil B, Lara N, Oteo J, et al. Isolates of beta-lactamase-negative ampicillin-resistant Haemophilus influenzae causing invasive infections in Spain remain susceptible to cefotaxime and imipenem. J Antimicrob Chemother. 2014;69:111–6. PubMed http://dx.doi.org/10.1093/jac/dkt324

10. Puig C, Grau I, Marti S, Tubau F, Calatayud L, Pallares R, et al. Clinical and molecular epidemiology of Haemophilus influenzae causing invasive disease in adult patients. PLoS ONE. 2014;9:e112711. PubMed http://dx.doi.org/10.1371/journal.pone.0112711

11. Resman F, Ristovski M, Ahl J, Forsgren A, Gilsdorf JR, Jasir A, et al. Invasive disease caused by Haemophilus influenzae in Sweden 1997–2009; evidence of increasing incidence and clinical burden of non-type b strains. Clin Microbiol Infect. 2011;17:1638–45. PubMed http://dx.doi.org/10.1111/j.1469-0691.2010.03417.x

12. Wang CC, Kuo HY, Chiang DH, Tsai CC, Lin ML, Chan YJ, et al. Invasive Haemophilus influenzae disease in adults in Taiwan. J Microbiol Immunol Infect. 2008;41:209–14. PubMed http://dx.doi.org/10.1111/j.1348-0421.2008.00033.x

13. Perdue DG, Bulkow LR, Gellin BG, Davidson M, Petersen KM, Singleton RJ, et al. Invasive Haemophilus influenzae disease in Alaskan residents aged 10 years and older before and after infant vaccination programs. JAMA. 2000;283:3089–94. PubMed http://dx.doi.org/10.1001/jama.283.23.3089

14. O’Neill JM, St Geme JW III, Cutter D, Adderson EE, Anyanwu J, Jacobs RF, et al. Invasive disease due to nontypeable Haemophilus influenzae among children in Arkansas. J Clin Microbiol. 2003;41:3064–9. PubMed http://dx.doi.org/10.1128/JCM.41.7.3064-3069.2003

15. Rubach MP, Bender JM, Mottice S, Hanson K, Weng HY, Korgenski K, et al. Increasing incidence of invasive Haemophilus influenzae disease in adults, Utah, USA. Emerg Infect Dis. 2011;17:1645–50. PubMed http://dx.doi.org/10.3201/eid1709.101991

16. Dworkin MS, Park L, Borchardt SM. The changing epidemiology of invasive Haemophilus influenzae disease, especially in persons >65 years old. Clin Infect Dis. 2007;44:810–6. PubMed http://dx.doi.org/10.1086/511861

17. MacNeil JR, Cohn AC, Farley M, Mair R, Baumbach J, Bennett N, et al. Current epidemiology and trends in invasive Haemophilus influenzae disease—United States, 1989–2008. Clin Infect Dis. 2011;53:1230–6. PubMed http://dx.doi.org/10.1093/cid/cir735

18. Livorsi DJ, Macneil JR, Cohn AC, Bareta J, Zansky S, Petit S, et al. Invasive Haemophilus influenzae in the United States, 1999–2008: epidemiology and outcomes. J Infect. 2012;65:496–504. PubMed http://dx.doi.org/10.1016/j.jinf.2012.08.005
